# Clinical relevance of circulating tumor cells in ovarian, fallopian tube and peritoneal cancer

**DOI:** 10.1007/s00404-020-05477-7

**Published:** 2020-03-06

**Authors:** Malgorzata Banys-Paluchowski, Tanja Fehm, Hans Neubauer, Peter Paluchowski, Natalia Krawczyk, Franziska Meier-Stiegen, Charlotte Wallach, Anna Kaczerowsky, Gerhard Gebauer

**Affiliations:** 1 Department of Gynecology and Obstetrics, Asklepios Klinik Barmbek, Rübenkamp 220, 22307 Hamburg, Germany; 2 Department of Obstetrics and Gynecology, Heinrich-Heine-University Düsseldorf, Moorenstr. 5, 40225 Düsseldorf, Germany; 3 Department of Gynecology and Obstetrics, Regio Klinik Pinneberg, Fahltskamp 74, 25421 Pinneberg, Germany; 4 Department of Gynecology and Obstetrics, Marienkrankenhaus Hamburg, Alfredstr. 9, 22087 Hamburg, Germany

**Keywords:** Ovarian cancer, Circulating tumor cell, Survival, Biomarker, Therapy monitoring

## Abstract

**Purpose:**

Presence of circulating tumor cells (CTCs) is associated with impaired clinical outcome in several solid cancers. Limited data are available on the significance of CTCs in gynaecological malignancies. The aims of the present study were to evaluate the dynamics of CTCs in patients with ovarian, fallopian tube and peritoneal cancer during chemotherapy and to assess their clinical relevance.

**Methods:**

43 patients with ovarian, fallopian tube and peritoneal cancer were included into this prospective study. Patients received chemotherapy according to national guidelines. CTC analysis was performed using the CellSearch system prior to chemotherapy, after three and six cycles.

**Results:**

In 26% of the patients, ≥ 1CTC per 7.5 ml of blood was detected at baseline (17% of patients with de novo disease, compared to 35% in recurrent patients). Presence of CTCs did not correlate with other factors. After three cycles of therapy, CTC positivity rate declined to 4.8%. After six cycles, no patient showed persistent CTCs. Patients with ≥ 1 CTC at baseline had significantly shorter overall survival and progression-free survival compared to CTC-negative patients (OS: median 3.1 months vs. not reached, *p* = 0.006, PFS: median 3.1 vs. 23.1 months, *p* = 0.005). When only the subgroup with newly diagnosed cancer was considered, the association between CTC status and survival was not significant (OS: mean 17.4 vs. 29.0 months, *p* = 0.192, PFS: 14.3 vs. 26.9 months, *p* = 0.085). Presence of ≥ 1 CTC after three cycles predicted shorter OS in the entire patient cohort (*p* < 0.001).

**Conclusions:**

Hematogenous tumor cell dissemination is a common phenomenon in ovarian, fallopian tube and peritoneal cancer. CTC status before start of systemic therapy correlates with clinical outcome. Chemotherapy leads to a rapid decline in CTC counts; further research is needed to evaluate the clinical value of CTC monitoring after therapy.

## Introduction

Ovarian cancer is the second most common gynaecological cancer and accounts for more deaths than any other cancer of the female reproductive system [1]. Despite optimal multivisceral cytoreductive surgery and standard platinum-based first-line chemotherapy, the majority of patients will suffer from a relapse within the first 2–3 years. Therefore, improved strategies to identify patients at risk for recurrence are urgently needed. In this context, blood-based biomarkers such as circulating tumor cells (CTCs) have emerged as a promising candidate.

Hematogenous dissemination of cancer cells shed by the primary tumor is a common phenomenon observed in several solid malignancies [2–4]. While blood-borne disease spread leading to development of distant metastases frequently occurs in entities such as breast, prostate and lung cancer, gynaecological tumors are more likely to show continuous spread within the abdominal cavity. Interestingly, based on clinical studies, isolated tumor cells can be detected in blood and bone marrow samples of patients with ovarian cancer with similar positivity rates as in breast cancer [5–7]. In a large pooled analysis of 495 patients with primary ovarian cancer disseminated tumor cells in bone marrow were detected in 27% of patients and predicted significantly shorter overall survival (OS) [6]. Since blood sampling is less invasive and allows serial measurements, the focus of translational research has shifted from disseminated tumor cells to CTCs in peripheral blood. Presence of two or more CTCs have already been shown to be associated with an unfavourable prognosis in relapsed ovarian cancer [7].

The aim of the present study was (1) to evaluate the prognostic relevance of CTCs at time of diagnosis and (2) to examine the dynamics of CTCs during chemotherapy in patients with ovarian, fallopian tube and peritoneal cancer.

## Methods

43 patients from two certified Gynaecological Cancer Centers were enrolled in this prospective, open-label, non-randomized study. 34 patients were diagnosed with ovarian cancer, five with fallopian tube cancer and four with primary peritoneal cancer. Patients were scheduled to receive chemotherapy in the first-line (*n* = 23) or higher-line (*n* = 20) setting. Further inclusion criteria were: age 18 years and older, and diagnosis of primary or relapsed ovarian, fallopian tube or peritoneal cancer. Blood samples were collected before start of a new line of chemotherapy chosen according to national and institutional standards as well as after three and six cycles of therapy. Response to therapy was evaluated according to institutional guidelines, mostly by CT scan and CA125 determination. Informed consent was obtained from all individual participants included in the study.

### Detection of CTCs

CTCs were detected using the CellSearch™ system (formerly Veridex LLC, NJ, USA, now Menarini Silicon Biosystems, Italy). Briefly, 7.5 ml peripheral blood were collected into CellSave Tubes and processed according to manufacturer’s instructions. The assay consists of an immunomagnetic enrichment step employing immunomagnetic beads coated with anti-epithelial cell adhesion molecule (EpCAM) antibody, followed by staining with several antibodies. A circulating tumor cell is defined as a CD45-negative cytokeratin-positive cell with a DAPI-stained nucleus. In the current study, CTC-positive patients were defined as those with at least one tumor cell per 7.5 ml blood.

### Statistical analysis

Chi-squared test were used to evaluate the relationship between CTC detection and clinical-pathological factors. In the survival analysis, following primary end points were considered: (1) death and (2) progression. Survival intervals were measured from the time of blood sampling to the time of death or of the first clinical, histological or radiographic diagnosis of progression. We constructed Kaplan–Meier curves and used the log-rank test to assess the univariate significance of the parameters. Cox regression analysis was used for multivariate analysis. All reported p-values are two-sided. *p* values ≤ 0.05 were considered significant. Statistical analysis was performed by SPSS (SPSS Inc., Chicago, IL, USA). The analysis was performed according to the REporting recommendations for tumor MARKer prognostic studies (REMARK) criteria on reporting of biomarkers [8]. The primary question was the prognostic impact of CTCs in the entire patient cohort.

## Results

### Patients’ characteristics

Clinical–pathological data of 43 patients enrolled in the study are summarized in Tables 1 and 2. Blood sample was collected at time of first diagnosis of malignant disease in 53% of patients, in the remaining 47% of cases at time of recurrent or progressive disease. The majority of patients had ovarian cancer (79%), followed by fallopian tube (12%) and peritoneal cancer (9%). Previous therapies received by patients with recurrent/progressive disease are shown in Table 3. Details regarding therapy administered during study are shown in Tables 3 and 4. Among patients with primary disease, all but one received primary debulking surgery and were scheduled for adjuvant systemic treatment in accordance with current national treatment guidelines. In one case (Patient 40, Table 4) with advanced disease and tumor rest > 2 cm, the patient refused further blood sampling and received neoadjuvant systemic therapy followed by secondary laparotomy with hyperthermic intraperitoneal chemotherapy (HIPEC) at another hospital. The BRCA status of the tumor has been assessed in 10 patients with recurrent/progressive disease and revealed a somatic BRCA1 mutation in one case. The remaining nine patients had BRCA-negative tumors.

**Table 1:** Distribution of the study patients according to circulating tumor cells in correlation to clinical-pathological characteristics

	Total	CTC positive at baseline *n* (%)	*p* value
Overall	43	11 (26%)	
Cancer origin			0.953
Ovarian cancer	34	9 (27%)	
Fallopian tube cancer	5	1 (20%)	
Primary peritoneal cancer	4	1 (25%)	
Disease setting			0.187
Newly diagnosed cancer	23	4 (17%)	
Recurrence	20	7 (35%)	
Histology			0.331
Serous high-grade	37	9 (24%)	
Serous low-grade	2	1 (50%)	
Endometrioid	2	0 (0%)	
Clear cell	1	0 (0%)	
Undifferentiated (G4)	1	1 (100%)

**Table 2:** Correlation of CTC status and established parameters in patients with newly diagnosed cancer

	Total	CTC positive at baseline *n* (%)	*p* value
T stage			0.191
T1-2	6	0 (0%)	
T3	17	4 (24%)	
FIGO stage			0.191
I–II	6	0 (0%)	
III–IV	17	4 (24%)	
Nodal status			0.825^b^
Node-negative	8	1 (13%)	
Node-positive	6	1 (17%)	
Unknown^a^	9	2 (22%)	
Residual tumor			0.106
No (macroscopic complete resection)	14	1 (7%)	
Yes	9	3 (33%)	
Histology			0.586
Serous high-grade	19	3 (16%)	
Serous low-grade	2	1 (50%)	
Endometrioid	1	0 (0%)	
Clear cell	1	0 (0%)	

^a^According to the national guidelines at time of surgical treatment the systematic lymphadenectomy was performed only when optimal cytoreduction has been achieved

^b^Analysis performed for patients with known nodal status

**Table 3:** Patients with recurrent/progressive disease at time of study enrollment: overview of therapies

Patient number	Disease setting during study	Previous systemic therapy	Surgical therapy in the current disease setting	Therapy during study
1	Third line	Carboplatin, paclitaxel, bevacizumab Carboplatin, gemcitabine, bevacizumab (within a clinical trial)	No	Carboplatin, pegylated liposomal doxorubicin
2	Second line	Carboplatin, paclitaxel, bevacizumab	Yes, debulking before start of second line therapy	Pegylated liposomal doxorubicin, trabectedin
3	Forth line	Carboplatin, paclitaxel Carboplatin, paclitaxel, bevacizumab Carboplatin, paclitaxel, maintenance therapy with olaparib	Yes, debulking before start of forth line therapy	Pegylated liposomal doxorubicin, trabectedin
4	Fifth line	Carboplatin, paclitaxel Carboplatin, gemcitabine Carboplatin, gemcitabine Carboplatin, gemcitabine	No	Pegylated liposomal doxorubicin, trabectedin
5	Second line	Carboplatin, paclitaxel, bevacizumab	No	Pegylated liposomal doxorubicin, trabectedin
6	Second line	Carboplatin, paclitaxel	No	Carboplatin
7	Second line	No systemic therapy administered after surgery	Yes, debulking before start of systemic therapy	Carboplatin
8	Second line	Carboplatin, paclitaxel, bevacizumab	Yes, debulking before start of second line therapy	Carboplatin, pegylated liposomal doxorubicin
9	Second line	Carboplatin, paclitaxel, bevacizumab	No	Carboplatin, pegylated liposomal doxorubicin
10	Second line	Carboplatin, paclitaxel, bevacizumab	No	Carboplatin, pegylated liposomal doxorubicin
11	Third line	Carboplatin, paclitaxel, bevacizumab Carboplatin, pegylated liposomal doxorubicin, bevacizumab (within a clinical trial)	No	Carboplatin, pegylated liposomal doxorubicin, bevacizumab
12	Third line	Carboplatin, paclitaxel Pegylated liposomal doxorubicin, trabectedin	No	None (best supportive care)
13	Second line	Carboplatin, paclitaxel, bevacizumab	No	Carboplatin, paclitaxel
14	Third line	Carboplatin, paclitaxel Pegylated liposomal doxorubicin, trabectedin	No	Carboplatin, paclitaxel
15	Second line	No systemic therapy administered after surgery (patient’s refusal)	No	Carboplatin, paclitaxel, bevacizumab
16	Fifth line	Carboplatin, paclitaxel Carboplatin, gemcitabine, bevacizumab Pegylated liposomal doxorubicin Topotecan	No	None (best supportive care)
17	Third line	Carboplatin, paclitaxel, bevacizumab Carboplatin, pegylated liposomal doxorubicin	No	Paclitaxel
18	Third line	Carboplatin, paclitaxel Pegylated liposomal doxorubicin	No	Topotecan
19	Fifth line	Carboplatin, paclitaxel Carboplatin, gemcitabine Pegylated liposomal doxorubicin, trabectedin Topotecan	No	Topotecan
20	Second line	Unknown (first-line therapy administered abroad)	No	None (best supportive care)

**Table 4:** Patients with primary disease at time of study enrollment: overview of therapies

Patient number	Therapy during study	Chemotherapy administered as planned
21	Carboplatin, paclitaxel, bevacizumab	Yes
22	Carboplatin, paclitaxel, bevacizumab	Yes
23	Carboplatin, paclitaxel, bevacizumab	Yes
24	Carboplatin, paclitaxel, bevacizumab	Yes
25	Carboplatin, paclitaxel, bevacizumab	Yes
26	1 cycle of carboplatin, paclitaxel, followed by 6 cycles of carboplatin weekly	No, switch to weekly monotherapy due to reduced performance status
27	Carboplatin, paclitaxel	Yes
28	None (best supportive care)	
29	Carboplatin, paclitaxel, bevacizumab	Yes
30	Carboplatin, paclitaxel, bevacizumab	Yes
31	Carboplatin, paclitaxel	Yes
32	Carboplatin, paclitaxel	Yes
33	Carboplatin, paclitaxel	Yes
34	Carboplatin, paclitaxel	Yes
35	Carboplatin, paclitaxel	Yes
36	None (best supportive care)	
37	Carboplatin weekly	No, therapy discontinuation due to adverse events after 3 cycles
38	Carboplatin, paclitaxel, bevacizumab	Yes
39	Carboplatin, paclitaxel	Yes
40	No therapy details available	No, study discontinuation after the first blood samples (patient’s request)
41	Carboplatin, paclitaxel, bevacizumab	Yes
42	Carboplatin, paclitaxel, bevacizumab	Yes
43	None (best supportive care)	

### Correlation of CTCs with clinical-pathological data

In 26% of patients at least one CTC per 7.5 ml of peripheral blood was detected at baseline (range 0–76, mean 2.84). Presence of CTC at time of diagnosis was not associated with the tumor origin and established prognostic factors such as tumor stage or nodal status. CTC status did not correlate with macroscopic tumor rest. At least one CTC was detected in 17% of patients with de novo disease, compared to 35% in recurrent patients, however this difference was not statistically significant (*p* = 0.187). After three cycles of systemic therapy, the CTC positivity rate declined to 4.8%; all patients with primary cancer were CTC-negative at this time point. After six cycles of therapy, no patient showed persistent CTCs.

### Survival analysis

**Fig. 1:**
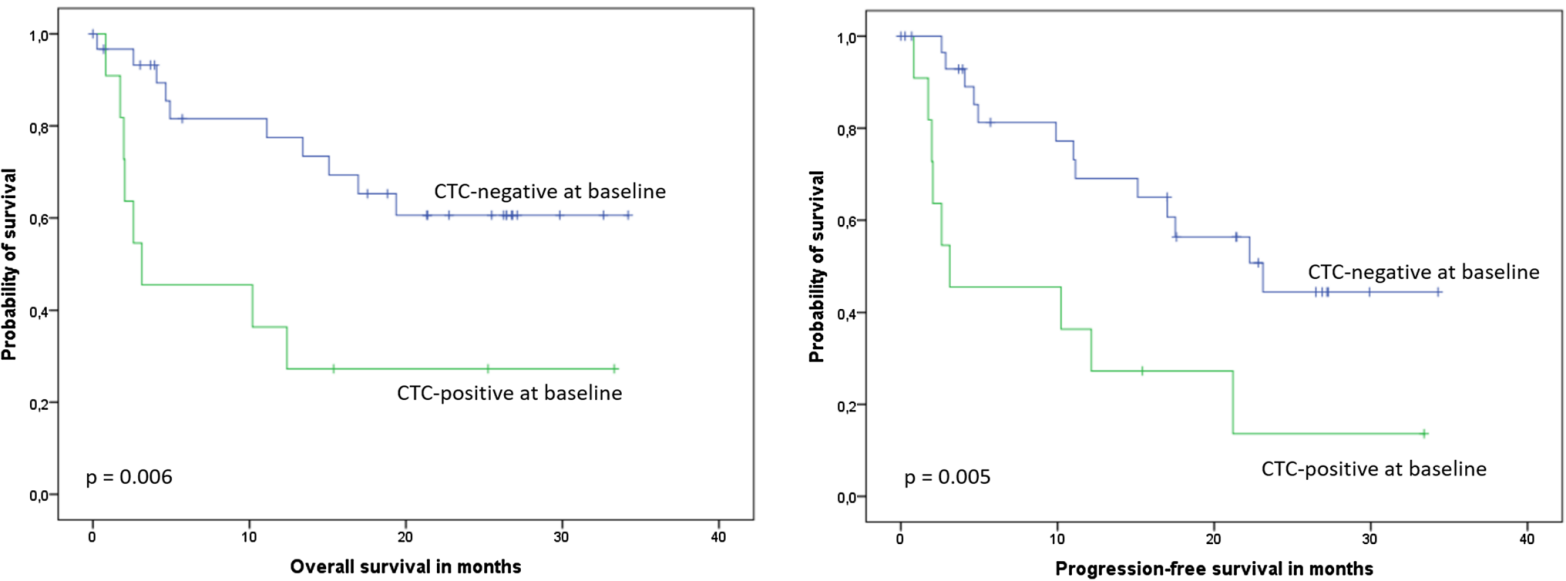
Kaplan–Meier plots of overall and progression-free survival according to CTC status in the entire patient cohort

**Fig. 2:**
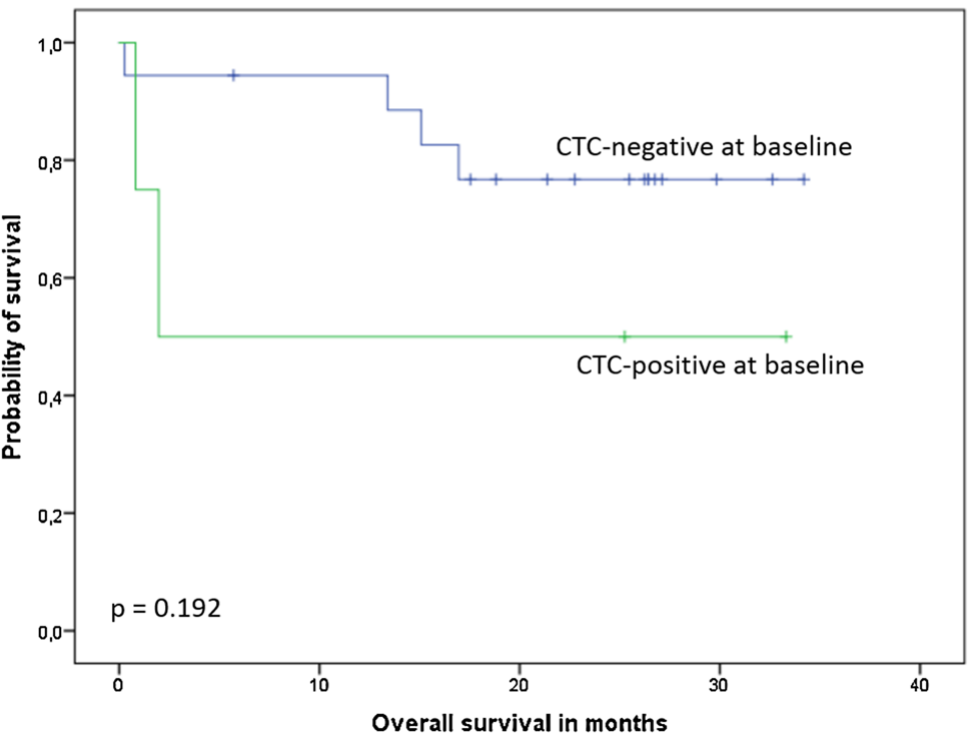
Kaplan–Meier plot of overall survival according to CTC status in the subgroup with primary non-relapsed cancer

After a median follow-up of 25 months (range 3–36 months), 18 patients died. Patients with at least one detectable CTC at baseline had significantly shorter OS compared to CTC-negative patients (mean OS 12.3 [95% CI 4.4–20.1] vs. 24.6 [19.7–29.4] months, median 3.1 [0.0–12.0] vs. not reached; *p* = 0.006, Fig. 1, Table 5). When only the subgroup with newly diagnosed cancer was considered, the association between CTC status and survival was not significant (mean 17.4 [1.7–33.1] vs. 29.0 [24.3–33.7] months, *p* = 0.192, Fig. 2, Table 5). Presence of at least one CTC after three cycles of systemic treatment predicted shorter OS in the entire patient cohort (mean OS 11.1 vs. 31.2 months, *p* < 0.001). In the entire cohort, CTC-positive patients at baseline had median progression-free survival of 3.1 months, compared to 23.1 months in CTC-negative patients (*p* = 0.005, Fig. 1, Table 5). In the multivariate analysis including CTC status, disease setting, histology and tumor rest, only the presence of CTCs significantly predicted reduced OS, while residual tumor and CTC status were the only independent factors predicting PFS (Table 6).

**Table 5:** Univariate analysis of CTC status and overall and progression-free survival according to disease setting

	Overall survival (months)		Progression-free survival (months)	
	CTC-positive vs. CTC-negative	*p* value	CTC-positive vs. CTC-negative	*p* value
Entire cohort (*n* = 43)	Mean 12.3 [95% CI 4.4—20.1] vs. 24.6 [19.7–29.4] Median: 3.1 [0.0–12.0] vs. NR	0.006	Mean 10.6 [3.8–17.4] vs. 22.1 [17.2–26.9] Median: 3.1 [0.0–12.0] vs. 23.1 [13.1–33.1]	0.005
Primary cancer (*n* = 23)	Mean 17.4 [1.7–33.1] vs. 29.0 [24.3–33.7] Median 1.9 vs. NR	0.192	Mean 14.3 [1.0–27.7] vs. 26.9 [22.2–31.6] Median 2.0 [0.0–21.9] vs. NR	0.085
Recurrent/progressive disease (*n* = 20)	Mean 6.8 [2.9–10.7] vs. 13.0 [6.3–19.7] Median 3.1 [1.8–4.5] vs. 11.1 [2.6–19.6]	0.169	Mean 6.8 [2.9–10.7] vs. 11.7 [5.5–17.9] Median 3.1 [1.8–4.5] vs. 4.9 [0.0–14.0]	0.251

*CI* confidence interval, *NR* not reached

**Table 6:** Multivariate analysis of overall and progression-free survival

Parameter	Overall survival			Progression-free survival		
	*p* value	Hazard ratio	95% CI	*p* value	Hazard ratio	95% CI
CTC status						
Positive vs. negative	0.018	3.439	1.23–9.61	0.002	4.389	1.72–11.20
Disease setting						
Primary vs. recurrent/progressive disease	0.618	0.666	0.14–3.30	0.327	2.083	0.48–9.03
Histology						
Serous high grade vs. other	0.591	1.420	0.40–5.09	0.649	1.298	0.42–3.99
Residual tumor						
No surgical therapy vs. macroscopic tumor rest vs. no tumor rest	0.167	2.116	0.73–6.14	0.003	5.003	1.71–14.65

*CI* confidence interval

## Discussion

In the present study, we demonstrated that hematogenous dissemination is a common phenomenon in patients with ovarian, fallopian tube and primary peritoneal cancer. Using the CellSearch assay, CTCs were detected in one-fourth of enrolled patients. While this finding might be at first surprising—giving the preference of these tumor entities for local tumor growth within abdominal cavity—it confirms our previously published data on the presence of disseminated tumor cells in bone marrow of patients with gynaecological malignancies [6]. In this prospective multicentre trial including 495 patients with primary ovarian cancer we reported a prevalence rate of disseminated tumor cells of 27%. Similar positivity rates were observed by others (Table 7) [9–13]. Since bone marrow sampling involves an invasive procedure, the research focus has shifted to examination of peripheral blood over the last two decades and an increasing body of evidence on CTCs in the blood of ovarian cancer patients is available. The largest study to date was conducted in relapsed ovarian cancer. Poveda et al. detected CTCs using the same assay as in our study (CellSearch) and reported a significantly reduced progression-free and overall survival in CTC-positive patients [7]. Interestingly, in contrast to other trials, Poveda et al. defined CTC-positivity as presence of two or more CTCs per 7.5 ml blood, so patients with one CTC were qualified as CTC-negative. Setting a specific cut-off value in case of CTC-based trials is common in other entities. For instance, in metastatic breast cancer several clinical trials used 5 CTCs per 7.5 ml blood as a threshold to differentiate between patients with favourable and unfavourable outcome [14–16], whereas 3 CTCs have been shown to be a more suitable cut-off value in metastatic colorectal cancer [17].

**Table 7:** Prevalence and prognostic relevance of circulating and disseminated tumor cells at time of diagnosis in patients with ovarian, fallopian tube and peritoneal cancer

Study	Tumor entity	Target cells / Assay	Positivity rate	Prognostic relevance
Our study	Primary and relapsed ovarian, fallopian tube and peritoneal cancer	CTCs CellSearch	26% (17% in primary, 25% in relapsed cancer)	OS, PFS^a,b^
Fehm, Banys et al. 2013 [6]	Primary ovarian cancer	DTCs ICC	27%	OS, PFS^b^
Poveda et al. 2011 [7]	Relapsed ovarian cancer	CTCs CellSearch	14% (defined as ≥ 2 CTCs per 10 ml blood)	OS, PFS
Banys et al. [5]^c^	Primary ovarian cancer	DTCs ICC	25%	DFS
Zhang et al. [22]	Primary ovarian cancer	CTCs Immunobeads, Multiplex-RT-PCR	90%	OS shorter in patients with EpCAM-positive CTCs
Braun et al. [12]^c^	Primary ovarian cancer	DTCs ICC	30%	DFS, DDFS^b^, OS
Marth et al. 2002 [10]	Primary ovarian cancer	CTCs Immunobeads	12%	n.s
		DTCs Immunobeads	21%	n.s
Schindlbeck et al. [11]	Primary ovarian cancer	DTCs ICC	23%	DDFS
Aktas et al. 2011 [9]	Primary ovarian cancer	CTCs Multiplex-RT-PCR (AdnaTest)	19%	OS
		DTCs ICC	35%	n.s
Chebouti et al. [13]	Primary ovarian cancer	DTCs ICC	42%	OS
Fan et al. [18]	Primary ovarian cancer	CTCs Immunofluorescence and cell invasion assay	61%	DFS
Sehouli et al. [19]	Primary ovarian cancer	CTCs ICC	n.a	n.s

*CTCs* circulating tumor cells, *DDFS* distant disease-free survival, *DFS* disease-free survival, *DTCs* disseminated tumor cells, *ICC* immunocytochemistry, *OS* overall survival, *n.s.* not significant, *PFS* progression-free survival

^a^Entire cohort, statistical significance in subgroups not reached

^b^Multivariate analysis

^c^Tthese cohorts were completely or partially included in the pooled analysis [6]

While the prognostic relevance of hematogenous tumor cell dissemination was confirmed in large trials in entities such as breast cancer, data regarding ovarian cancer are still limited. In the present study presence of at least one CTC was associated with worse PFS and OS in the entire cohort. When patients with primary and relapsed cancer were considered as separate subgroups, the correlation was not significant. However, this trial was not powered for subgroup analysis. Similar results have been reported by several other studies. Positive CTC status, defined as ≥ 2 CTCs per 7.5 ml blood, predicted shorter progression-free survival and OS in patients with relapsed ovarian cancer [7]. An association with OS or disease-free survival has been reported by two smaller studies conducted in primary ovarian cancer as well [9, 18]. However, a correlation with survival has not been shown by others, so far [10, 19]. Evidence is clearer with respect to tumor cell detection in the bone marrow: in the pooled analysis of individual patients data from three centers presence of disseminated tumor cells significantly predicted reduced survival [6]. Several hypotheses were discussed as to the biological fate of the single tumor cells. While we cannot exclude the possibility that CTCs and disseminated tumor cells are solely an epiphenomenon of current tumor load, the available data suggest that their role is beyond being just a by-product without their own clinical relevance. Since patients with ovarian carcinoma rarely develop secondary bone metastases, bone marrow seems to serve as a temporary “compartment” for disseminated tumor cells, where they might stay dormant for prolonged periods of time [20, 21]. Subsequently, they might be able to leave their homing site and cause metastatic growth or locoregional recurrence [6]. Hypothetically, these single cells might also be able to re-populate the abdominal cavity, where they encounter a microenvironment suitable to support ovarian cancer growth.

Possibly, not only the presence of CTCs, but their expression profiles may predict the clinical potential. Zhang et al. examined blood samples from 109 patients with newly diagnosed ovarian cancer using Multiplex-RT-PCR based on the detection of six cancer-related genes [22]. While this assay yielded very high CTC detection rates of 90%, the survival analysis showed that only EpCAM positivity of CTCs predicted shorter OS. Interestingly, the CellSearch system, used in our study, includes an enrichment step based on anti-EpCAM antibodies. For that reason, CTCs detected by this assay are more likely to express EpCAM that those detected by other methods (Table 4).

Although the majority of patients with primary ovarian carcinoma initially responds to (neo)adjuvant platinum-based chemotherapy, most will relapse following the state-of-the-art treatment [23]. Therefore, strategies for identification of patients at high risk for relapse early during first-line therapy are urgently needed. In our study, the CTC positivity rate declined rapidly during treatment and no patient showed CTCs at the end of sixth cycle of chemotherapy. In the study by Zhang et al., CTC counts decreased during adjuvant and neoadjuvant therapy as well [22]. Interestingly, peripheral blood was obtained both before and 7–14 days after surgery and a rapid increase in CTC counts has been observed between these two time points. Since the baseline blood sample in our study was collected after surgery, we do not know whether such a decline could be observed using the CellSearch detection system as well. In contrast, Aktas et al. evaluated blood samples from primary ovarian cancer patients obtained before surgery in 86 and/or after adjuvant chemotherapy in 70 cases using the RT-PCR-based AdnaTest and found higher CTC positivity rate after chemotherapy (27% vs. 19%, respectively) [9]. Positive CTC status correlated with shorter OS, independent of the time point of blood sampling (*p* = 0.0054 before surgery and *p* = 0.047 after chemotherapy).

## Conclusions

In this prospective translational study, we show that hematogenous tumor cell dissemination is a common phenomenon in ovarian, fallopian tube and primary peritoneal carcinoma and is not restricted to patients with high-grade or node-positive disease. With regard to the clinical relevance of this phenomenon, CTC detection before start of adjuvant treatment significantly predicted shorter OS and PFS. However, since CTC counts declined rapidly during systemic therapy, this approach does not seem likely to identify patients at particular risk of relapse. Future research is required to fully understand the potential of CTC detection and characterization in patients with these tumor entities.

## Data Availability

The datasets generated during the current study are available from the corresponding author on reasonable request.

## Abbreviations

CTC: Circulating tumor cell
DDFS: Distant disease-free survival
DFS: Disease-free survival
DTC: Disseminated tumor cell
EpCAM: Epithelial cell adhesion molecule
ICC: Immunocytochemistry
OS: Overall survival
PFS: Progression-free survival
REMARK: REporting recommendations for tumor MARKer prognostic studies
